# Automated real time constant-specificity surveillance for disease outbreaks

**DOI:** 10.1186/1472-6947-7-15

**Published:** 2007-06-13

**Authors:** Shannon C Wieland, John S Brownstein, Bonnie Berger, Kenneth D Mandl

**Affiliations:** 1 Department of Mathematics, Massachusetts Institute of Technology, Cambridge, MA 02139-4307, USA; 2 Children's Hospital Informatics Program at the Harvard MIT Division of Health Sciences and Technology, Children's Hospital Boston, Boston, MA 02115, USA; 3 Computer Science and Artificial Intelligence Laboratory, M.I.T., Massachusetts Avenue, Cambridge, MA 02139-4307, USA; 4 Harvard Medical School, Shattuck Street, Boston, MA 02115-6092, USA

## Abstract

**Background:**

For real time surveillance, detection of abnormal disease patterns is based on a difference between patterns observed, and those predicted by models of historical data. The usefulness of outbreak detection strategies depends on their specificity; the false alarm rate affects the interpretation of alarms.

**Results:**

We evaluate the specificity of five traditional models: autoregressive, Serfling, trimmed seasonal, wavelet-based, and generalized linear. We apply each to 12 years of emergency department visits for respiratory infection syndromes at a pediatric hospital, finding that the specificity of the five models was almost always a non-constant function of the day of the week, month, and year of the study (*p *< 0.05). We develop an outbreak detection method, called the expectation-variance model, based on generalized additive modeling to achieve a constant specificity by accounting for not only the expected number of visits, but also the variance of the number of visits. The expectation-variance model achieves constant specificity on all three time scales, as well as earlier detection and improved sensitivity compared to traditional methods in most circumstances.

**Conclusion:**

Modeling the variance of visit patterns enables real-time detection with known, constant specificity at all times. With constant specificity, public health practitioners can better interpret the alarms and better evaluate the cost-effectiveness of surveillance systems.

## Background

The release of anthrax in 2001, the Severe Acute Respiratory Syndrome (SARS) outbreaks in China, Hong Kong and Toronto in 2002, and the emergence of new diseases such as West Nile virus have underscored the need for automated, real-time detection of outbreaks. Several such detection systems have been deployed in recent years at the hospital [1,2], city [3-5], regional [6-8] and national [9-11] levels. Many systems use time series algorithms to detect aberrant conditions, such as CuSUM [12-14], variants of the Serfling method [3], multiresolution wavelet-based models [15], and trimmed seasonal models [16].

An outcome of any of these statistical methods – whether or not there is an alarm on any given day – is uninformative without an estimate of the likelihood that an alarm signals a true outbreak. This likelihood depends in part on the specificity of the detection method, equal to the proportion of non-outbreak days for which no alarm is raised. The specificity is related to the false alarm rate by the simple equation

false alarm rate = 1 - specificity.

Even small changes in the specificity of the detection method may have a large impact on the likelihood of a true outbreak. Despite the importance of knowing the specificity, analysis of the specificity of outbreak detection algorithms has been rudimentary, and it is common practice to report one average value of specificity that is assumed to reflect the true specificity on any day of the year or week. Implicit in this is the assumption that the specificity is constant as a function of time. If this assumption is incorrect – if instead the specificity of an outbreak detection system is a function of time that deviates significantly from its average value – then on any given day, a public health practitioner cannot know the specificity of the system or the related probability that there is a disease outbreak, and therefore cannot respond appropriately to alarms.

The sensitivity of a method, or proportion of outbreaks detected, is negatively associated with its specificity. Unlike the specificity, however, it cannot be evaluated from non-outbreak data. This is because in addition to its dependence on the specificity, it also depends on the characteristics of an outbreak, including its duration and magnitude. Hence the trade-off between sensitivity and specificity must be carefully considered in the context of the outbreak type of interest to ensure that both fall in a useful range.

We sought to characterize changes in the specificity of alarms produced by standard time series outbreak detection methods as a function of time. We further explored how these changes affect the sensitivity of detection methods to several outbreak types. We introduced a statistical technique that allows us to model properties of time series not captured by traditional models, developing an outbreak detection strategy with constant specificity that may be used by public health practitioners for biosurveillance.

## Methods

### Data

Data were collected retrospectively in the emergency department (ED) of an urban pediatric tertiary care teaching hospital. All patients with respiratory presenting complaints seen in the ED between August 1, 1992 and July 30, 2004 were included in the study. The data were divided into a six-year training period, and a test period consisting of the final six years. ED chief complaints were selected at triage from among a constrained list, and classified as respiratory or non-respiratory using a previously validated method [17]. The study was approved by the institutional review board.

**Figure 1:**
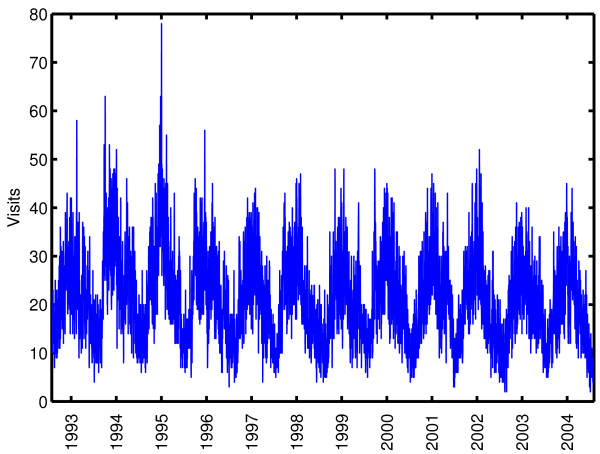
**Emergency department visits for respiratory presenting complaints, August 1, 1992 – July 30, 2004**. Daily time series showing the number of patients presenting with respiratory complaints to the emergency department during a 12 year period.

During the study period, approximately 137 patients were seen each day in the ED. The number of daily visits for respiratory complaints varied from 2 to 78. The mean number of respiratory visits was 21.05, and the standard deviation was 9.03 (see figure 1). These data and other hospital visit data time series have previously been shown to depend significantly on the day of the week and the season of the year [16,18-20].

### Time series algorithms

We implemented five traditional time series models used for outbreak detection: a simple autoregressive model, a Serfling model, the trimmed seasonal model, a wavelet-based model, and a generalized linear model. In addition, we introduced a model of both the expectation and the variance based on generalized additive modeling techniques. The input to each algorithm was a time series of historical daily ED respiratory visit counts, and each returned a threshold number of visits for the day immediately following the historical period. An alarm occurred when the actual number of visits exceeded the threshold.

### Autoregressive model

The autoregressive model predicted the number of ED respiratory visits using linear regression on the number of visits during the previous seven days:

Et=a0+∑k=17ak⋅Vt−k,
 MathType@MTEF@5@5@+=feaafiart1ev1aaatCvAUfKttLearuWrP9MDH5MBPbIqV92AaeXatLxBI9gBaebbnrfifHhDYfgasaacH8akY=wiFfYdH8Gipec8Eeeu0xXdbba9frFj0=OqFfea0dXdd9vqai=hGuQ8kuc9pgc9s8qqaq=dirpe0xb9q8qiLsFr0=vr0=vr0dc8meaabaqaciaacaGaaeqabaqabeGadaaakeaacqWGfbqrdaWgaaWcbaGaemiDaqhabeaakiabg2da9iabdggaHnaaBaaaleaacqaIWaamaeqaaOGaey4kaSYaaabCaeaacqWGHbqydaWgaaWcbaGaem4AaSgabeaakiabgwSixlabdAfawnaaBaaaleaacqWG0baDcqGHsislcqWGRbWAaeqaaaqaaiabdUgaRjabg2da9iabigdaXaqaaiabiEda3aqdcqGHris5aOGaeiilaWcaaa@4577@

where *E*_*t *_is the predicted number of visits on day *t*, *V*_*t*-*k *_is the actual number of visits on day *t *- *k*, and the coefficients *a*_*k *_were fitted by least squares regression using training data.

### Serfling method

The Serfling method and its variants have been extensively used for surveillance of influenza and other diseases [3,21,22]. Our implementation modeled the number of daily visits using linear regression on sine and cosine terms having yearly periodicities to capture seasonal effects, categorical variables for the day of week, and linear and quadratic terms. Under this model, the predicted number of visits on day *t *was

Et=∑k=06ak⋅δk,dow(t)+a7+a8⋅t+a9⋅t2+a10⋅sin⁡(2π⋅doy(t)365)+a11⋅cos⁡(2π⋅doy(t)365),
 MathType@MTEF@5@5@+=feaafiart1ev1aaatCvAUfKttLearuWrP9MDH5MBPbIqV92AaeXatLxBI9gBaebbnrfifHhDYfgasaacH8akY=wiFfYdH8Gipec8Eeeu0xXdbba9frFj0=OqFfea0dXdd9vqai=hGuQ8kuc9pgc9s8qqaq=dirpe0xb9q8qiLsFr0=vr0=vr0dc8meaabaqaciaacaGaaeqabaqabeGadaaakeaacqWGfbqrdaWgaaWcbaGaemiDaqhabeaakiabg2da9maaqahabaGaemyyae2aaSbaaSqaaiabdUgaRbqabaGccqGHflY1iiGacqWF0oazdaWgaaWcbaGaem4AaSMaeiilaWIaeeizaqMaee4Ba8Maee4DaCNaeiikaGIaemiDaqNaeiykaKcabeaaaeaacqWGRbWAcqGH9aqpcqaIWaamaeaacqaI2aGna0GaeyyeIuoakiabgUcaRiabdggaHnaaBaaaleaacqaI3aWnaeqaaOGaey4kaSIaemyyae2aaSbaaSqaaiabiIda4aqabaGccqGHflY1cqWG0baDcqGHRaWkcqWGHbqydaWgaaWcbaGaeGyoaKdabeaakiabgwSixlabdsha0naaCaaaleqabaGaeGOmaidaaOGaey4kaSIaemyyae2aaSbaaSqaaiabigdaXiabicdaWaqabaGccqGHflY1cyGGZbWCcqGGPbqAcqGGUbGBdaqadaqaamaalaaabaGaeGOmaiJae8hWdaNaeyyXICTaeeizaqMaee4Ba8MaeeyEaKNaeiikaGIaemiDaqNaeiykaKcabaGaeG4mamJaeGOnayJaeGynaudaaaGaayjkaiaawMcaaiabgUcaRiabdggaHnaaBaaaleaacqaIXaqmcqaIXaqmaeqaaOGaeyyXICTagi4yamMaei4Ba8Maei4Cam3aaeWaaeaadaWcaaqaaiabikdaYiab=b8aWjabgwSixlabbsgaKjabb+gaVjabbMha5jabcIcaOiabdsha0jabcMcaPaqaaiabiodaZiabiAda2iabiwda1aaaaiaawIcacaGLPaaacqGGSaalaaa@9219@

where dow(*t*) is the day of the week from 0 to 6, doy(*t*) is the day of the year from 1 to 365, and the Kronecker delta function *δ*_*x*,*y *_is equal to 1 when *x *= *y *and 0 otherwise. To calculate the day of the year during leap years, each day after February 28 was treated as though it occurred on the previous day.

### Trimmed seasonal model

The trimmed seasonal model is used in the AEGIS system [23] for statewide real-time population health monitoring, and was implemented as previously described [16]. Beginning with training set data, the average number of visits was calculated and subtracted from the data. From this, the average for each day of the week was calculated and again subtracted. To remove seasonal effects, the average for the day of the year was calculated after excluding the highest and lowest 25% of values for each day of the year, and again subtracted from the data. A first-order autoregressive, first-order moving average (ARMA) model was then fitted to the errors. The predicted number of visits *E*_*t *_was calculated by summing the overall average, the average for the day of the week, the average for the day of the year, and the ARMA prediction for day *t*.

### Wavelet model

The wavelet-based model was patterned after the wavelet anomaly detector developed by Zhang et al. [15]. The method used the number of daily visits in a training set, *V*_1_, *V*_2_, ..., *V*_*t*-1_, to produce a prediction for day *t*. It consisted of the following steps:

1. A low-frequency wavelet component of the visit signal having periodicity of more than 32 days was calculated. This period was selected by Zhang et al. because it removes seasonal effects while preserving higher-frequency information, and because it is a power of 2, which is mathematically convenient for wavelet analysis. We used the Haar wavelet in our implementation of the model [24].

2. This low-frequency baseline was subtracted from the original signal, producing a residual for each day in the training set.

3. The predicted number of visits on day *t *was the value of the low-frequency component on the previous day.

Daily alarm thresholds for the autoregressive, Serfling, trimmed seasonal, and wavelet-based models were calculated as the sum of the expected number of visits and a multiple *λ *of the standard deviation of the model residuals on the historical training data. The value *λ *of was an adjustable parameter that affected the specificity of each model.

### Generalized linear model

The generalized linear model consisted of a Poisson distribution function, an identity link function, and a linear predictor that included day of the week, month of the year, holiday and linear trend terms:

Et=β0+∑k=05βk+1⋅δk,dow(t)+∑k=011βk+6⋅δk,moy(t)+β18Iholiday(t)+β19t,
 MathType@MTEF@5@5@+=feaafiart1ev1aaatCvAUfKttLearuWrP9MDH5MBPbIqV92AaeXatLxBI9gBaebbnrfifHhDYfgasaacH8akY=wiFfYdH8Gipec8Eeeu0xXdbba9frFj0=OqFfea0dXdd9vqai=hGuQ8kuc9pgc9s8qqaq=dirpe0xb9q8qiLsFr0=vr0=vr0dc8meaabaqaciaacaGaaeqabaqabeGadaaakeaacqWGfbqrdaWgaaWcbaGaemiDaqhabeaakiabg2da9GGaciab=j7aInaaBaaaleaacqaIWaamaeqaaOGaey4kaSYaaabCaeaacqWFYoGydaWgaaWcbaGaem4AaSMaey4kaSIaeGymaedabeaakiabgwSixlab=r7aKnaaBaaaleaacqWGRbWAcqGGSaalcqqGKbazcqqGVbWBcqqG3bWDcqGGOaakcqWG0baDcqGGPaqkaeqaaaqaaiabdUgaRjabg2da9iabicdaWaqaaiabiwda1aqdcqGHris5aOGaey4kaSYaaabCaeaacqWFYoGydaWgaaWcbaGaem4AaSMaey4kaSIaeGOnaydabeaakiabgwSixlab=r7aKnaaBaaaleaacqWGRbWAcqGGSaalcqqGTbqBcqqGVbWBcqqG5bqEcqGGOaakcqWG0baDcqGGPaqkaeqaaOGaey4kaSIae8NSdi2aaSbaaSqaaiabigdaXiabiIda4aqabaGccqWGjbqsdaWgaaWcbaGaeeiAaGMaee4Ba8MaeeiBaWMaeeyAaKMaeeizaqMaeeyyaeMaeeyEaKhabeaakiabcIcaOiabdsha0jabcMcaPiabgUcaRiab=j7aInaaBaaaleaacqaIXaqmcqaI5aqoaeqaaOGaemiDaqNaeiilaWcaleaacqWGRbWAcqGH9aqpcqaIWaamaeaacqaIXaqmcqaIXaqma0GaeyyeIuoaaaa@81FF@

where dow(*t*) and *δ*_*x*,*y *_are described in equation 2, moy(*t*) is the month from 1 (January) to 12 (December), and *I*_holiday_(*t*) is an indicator function equal to 1 if day *t *is a holiday, and 0 otherwise. An alarm sounded if the value of the cumulative distribution function of a Poisson random variable with mean *E*_*t *_exceeded the desired specificity. This model was found by Jackson et al. [18] to have superior sensitivity to a variety of outbreak types compared to several control-chart and exponential weighted moving average models.

### Expectation-variance model

In addition, we developed and implemented a novel method for outbreak detection that captures changes in the ED visit standard deviation, as well as in the expected number of visits. In contrast to previous surveillance models, which assumed that the variance is constant or proportional to the mean, it did not assume a functional form for the variance. Instead, the dependence of both the mean number of visits and the variance was modeled explicitly. In other applications, several statisticians have modeled the variance as a function of the same or additional covariates used to model the mean using iterative successive relaxation procedures (see, for example, [25] and [26]). We employed a simplified procedure involving two distinct models: an expectation model of the daily expected number *E*_*t *_of respiratory ED visits, and a variance model of the daily variance σt2
 MathType@MTEF@5@5@+=feaafiart1ev1aaatCvAUfKttLearuWrP9MDH5MBPbIqV92AaeXatLxBI9gBaebbnrfifHhDYfgasaacH8akY=wiFfYdH8Gipec8Eeeu0xXdbba9frFj0=OqFfea0dXdd9vqai=hGuQ8kuc9pgc9s8qqaq=dirpe0xb9q8qiLsFr0=vr0=vr0dc8meaabaqaciaacaGaaeqabaqabeGadaaakeaaiiGacqWFdpWCdaqhaaWcbaGaemiDaqhabaGaeGOmaidaaaaa@3106@ of respiratory ED visits. The number of daily visits is then modeled as a Gaussian with mean *E*_*t *_and variance σt2
 MathType@MTEF@5@5@+=feaafiart1ev1aaatCvAUfKttLearuWrP9MDH5MBPbIqV92AaeXatLxBI9gBaebbnrfifHhDYfgasaacH8akY=wiFfYdH8Gipec8Eeeu0xXdbba9frFj0=OqFfea0dXdd9vqai=hGuQ8kuc9pgc9s8qqaq=dirpe0xb9q8qiLsFr0=vr0=vr0dc8meaabaqaciaacaGaaeqabaqabeGadaaakeaaiiGacqWFdpWCdaqhaaWcbaGaemiDaqhabaGaeGOmaidaaaaa@3106@. Both components are generalized additive models (GAM's): nonparametric extensions of linear regression models having several variants depending on the choice of smoothing technique, the procedure used to find estimates of the nonparametric functions for multivariate models, and the number of degrees of freedom for each covariate [27,28].

The GAM of the expectation accepted historical daily visit counts as input, and modeled them as a function of linear time to capture a long-term trend, the day of the year to account for seasonal trends, and the day of the week:

*E*_*t *_= *f*_trend_(*t*) + *f*_doy_(doy(*t*)) + *f*_dow_(dow(*t*)).

No smoothing was performed for the day-of-week term, since many replicates were available for each day of the week. A Gaussian kernel smoother was used for the trend term, and a Gaussian kernel smoother with circular boundaries was used for the day-of-year term since the day is a periodic covariate. Although a Gaussian was selected for its ease of interpretation, in general the choice of kernel function has little effect on the model compared to the choice of bandwidth [27]. Optimal bandwidths of the two Gaussian smoothers were estimated by a two-step procedure. First, to optimize the bandwidth of the day-of-year Gaussian, the mean predictive squared error (PSE) on a training set consisting of the first six years of ED visit data was calculated for a range of bandwidths using 10-fold cross-validation for a model containing only the day-of-week and day-of-year covariates. The bandwidth minimizing the mean PSE was chosen, corresponding to a Gaussian distribution with a standard deviation of five days. Next, the bandwidth of the kernel used for the trend term was chosen by using 10-fold cross-validation to estimate the mean PSE on the training set of a model containing all three covariates for a range of trend bandwidths, using the previously determined optimal bandwidth of the day-of-year kernel. The minimizing bandwidth was again chosen, corresponding to a standard deviation of eight days. Because the model contained multiple nonparametric functions, an iterative backfitting procedure was used to estimate each until the model converged [27].

The residuals of the expectation GAM on the historical data were squared and used as the input to the variance GAM. This GAM was also a function of linear time, day-of-year, and day-of-week variables:

σt2=gtrend(t)+gdoy(doy(t))+gdow(dow(t)).
 MathType@MTEF@5@5@+=feaafiart1ev1aaatCvAUfKttLearuWrP9MDH5MBPbIqV92AaeXatLxBI9gBaebbnrfifHhDYfgasaacH8akY=wiFfYdH8Gipec8Eeeu0xXdbba9frFj0=OqFfea0dXdd9vqai=hGuQ8kuc9pgc9s8qqaq=dirpe0xb9q8qiLsFr0=vr0=vr0dc8meaabaqaciaacaGaaeqabaqabeGadaaakeaaiiGacqWFdpWCdaqhaaWcbaGaemiDaqhabaGaeGOmaidaaOGaeyypa0Jaem4zaC2aaSbaaSqaaiabbsha0jabbkhaYjabbwgaLjabb6gaUjabbsgaKbqabaGccqGGOaakcqWG0baDcqGGPaqkcqGHRaWkcqWGNbWzdaWgaaWcbaGaeeizaqMaee4Ba8MaeeyEaKhabeaakiabcIcaOiabbsgaKjabb+gaVjabbMha5jabcIcaOiabdsha0jabcMcaPiabcMcaPiabgUcaRiabdEgaNnaaBaaaleaacqqGKbazcqqGVbWBcqqG3bWDaeqaaOGaeiikaGIaeeizaqMaee4Ba8Maee4DaCNaeiikaGIaemiDaqNaeiykaKIaeiykaKIaeiOla4caaa@5DBB@

The Gaussian smoothers were chosen to minimize the PSE on the training data set using the same procedure as above. The optimal smoothers corresponded to Gaussian distributions with standard deviations of 6 and 253 days for the day-of-year and trend terms, respectively.

To set the alarm threshold for a given day, a composite expectation-variance model consisting of the two GAM's was trained on the previous six years of data. The alarm threshold for the next day was calculated as the sum of the expected number of ED visits, as predicted by the expectation GAM, and a multiple *λ *of the expected standard deviation of ED visits, as predicted by the variance GAM:

*A*_*t *_= *E*_*t *_+ *λ*·*σ*_*t*_

=ftrend(t)+fdoy(doy(t))+fdow(dow(t))+λ⋅gtrend(t)+gdoy(doy(t))+gdow(dow(t)).
 MathType@MTEF@5@5@+=feaafiart1ev1aaatCvAUfKttLearuWrP9MDH5MBPbIqV92AaeXatLxBI9gBaebbnrfifHhDYfgasaacH8akY=wiFfYdH8Gipec8Eeeu0xXdbba9frFj0=OqFfea0dXdd9vqai=hGuQ8kuc9pgc9s8qqaq=dirpe0xb9q8qiLsFr0=vr0=vr0dc8meaabaqaciaacaGaaeqabaqabeGadaaakeaacqGH9aqpcqWGMbGzdaWgaaWcbaGaeeiDaqNaeeOCaiNaeeyzauMaeeOBa4MaeeizaqgabeaakiabcIcaOiabdsha0jabcMcaPiabgUcaRiabdAgaMnaaBaaaleaacqqGKbazcqqGVbWBcqqG5bqEaeqaaOGaeiikaGIaeeizaqMaee4Ba8MaeeyEaKNaeiikaGIaemiDaqNaeiykaKIaeiykaKIaey4kaSIaemOzay2aaSbaaSqaaiabbsgaKjabb+gaVjabbEha3bqabaGccqGGOaakcqqGKbazcqqGVbWBcqqG3bWDcqGGOaakcqWG0baDcqGGPaqkcqGGPaqkcqGHRaWkiiGacqWF7oaBcqGHflY1daGcaaqaaiabdEgaNnaaBaaaleaacqqG0baDcqqGYbGCcqqGLbqzcqqGUbGBcqqGKbazaeqaaOGaeiikaGIaemiDaqNaeiykaKIaey4kaSIaem4zaC2aaSbaaSqaaiabbsgaKjabb+gaVjabbMha5bqabaGccqGGOaakcqqGKbazcqqGVbWBcqqG5bqEcqGGOaakcqWG0baDcqGGPaqkcqGGPaqkcqGHRaWkcqWGNbWzdaWgaaWcbaGaeeizaqMaee4Ba8Maee4DaChabeaakiabcIcaOiabbsgaKjabb+gaVjabbEha3jabcIcaOiabdsha0jabcMcaPiabcMcaPaWcbeaakiabc6caUaaa@891E@

The value of *λ *was an adjustable model parameter.

All models were implemented using the Matlab software package, Version 7.0.1 [29]. The Matlab system identification, statistics and wavelet toolboxes were used for the wavelet, generalized linear, and expectation-variance models.

### Model predictions based on historical data

We used the expectation-variance model to generate alarm thresholds for each day during the test period from August 1, 1998 to July 30, 2004, which comprised the last six years of historical data. All of the available data could not be used for testing because a training period was required. To predict each threshold, the model was trained on the previous six years of data, ending the day before the day to be predicted, and was blind to the actual number of ED visits on the prediction day. The backfitting procedures to estimate the model successfully converged for each day of the study period. The model predictions for both the expected number of patients and the variance were always positive numbers throughout the study period. The average absolute predictive error was approximately four patients during the study period.

For each day, an alarm threshold was produced for each desired outbreak detection specificity between 0.01 and 0.99 in 0.01 increments. This was achieved by varying the threshold parameter *λ *appropriately. For example, to generate an alarm threshold with specificity *s *on day T, the model was trained on the historical visit data, *V*_*T*-2191_, ..., *V*_*T*-1_. This generated model estimates for the expected number of visits for each day, *E*_*T*-2191_, ..., *E*_*T*-1_, *E*_*T*_, as well as estimates for the expected standard deviation of visits, *σ*_*T*-2191_, ..., *σ*_*T*-1_, *σ*_*T*_. The parameter *λ *was chosen so that the fraction of historical days for which the Z-score was at most *λ *was as close as possible to the desired specificity *s*. That is, *λ *was chosen to have the property that

#{*t *: *T *- 2191 ≤ *t *≤ *T *- 1 and *V*_*t *_- *E*_*t *_≤ *λ*·*σ*_*t*_} ≈ 2191·*s*.

The predicted threshold for day T was *E*_*T *_+ *λ*·*σ*_*T*_.

Alarm thresholds for each day of the test period and each desired specificity were similarly calculated for the autoregressive, Serfling, trimmed seasonal, and wavelet models. The alarm threshold for the generalized linear model was the largest integer *A*_*t *_for which the cumulative distribution function of a Poisson random variable with mean *E*_*t *_was at most *s*. With the exception of wavelet model thresholds, all alarm thresholds were calculated using the six years of visit data immediately preceding the prediction day. The wavelet model requires a training period having length equal to a power of two, so 2048 days of training data were used.

### Detecting variability in the specificity

To determine whether a given model at a particular mean specificity had constant specificity as a function of the day of the week, we tabulated the proportion of alarm and non-alarm days at that mean specificity by day of the week. A chi-square analysis was performed under the null hypothesis that all days of the week had an equal fraction of alarm days. A *p*-value less than 0.05 indicated that the specificity was dependent on the day of the week. To determine whether the specificity was constant as a function of month and year, we performed similar chi-square analyses after tallying alarm days by month of the year and by calendar year of the study, respectively.

### Simulated outbreaks

In order to ascertain the sensitivity of the models to outbreaks, we superimposed three synthetic outbreaks on the test data set: a flat outbreak of five additional patients per day for seven days, a linear outbreak which increased from one to five patients over five days, and a spike outbreak of 10 additional patients in one day. For each model, each outbreak type, and each day of the test period, we created a new semisynthetic data set by adding an outbreak beginning on that day to the original data set. We then made an alarm threshold prediction for each of the outbreak days, and for each desired specificity between 0.01 and 0.99, based on training using the semisynthetic data set.

### Estimating sensitivity, specificity, and timeliness of detection

The actual mean specificity for one model at each desired input specificity was determined by running the model on the historical data set. Specificity was estimated by calculating the fraction of days without alarms for each day of the week, month of the year, or calendar year. Sensitivity calculations used the results of applying each of the models to the semisynthetic data sets. The sensitivity was calculated as the fraction of outbreaks for which there was at least one alarm day. Exact 95 percent binomial confidence intervals were calculated for each estimate of sensitivity and specificity. Timeliness of detection was evaluated for each method by calculating the mean lag in days between the start of a flat outbreak and the first alarm sounded. Missed outbreaks, for which no alarms were sounded on any day of the outbreak, were excluded from timeliness calculations. An alarm sounding on the first outbreak day corresponded to a lag of zero. Timeliness calculations were calculated at the benchmark specificity values of 0.85 and 0.97.

### Comparing outbreak detection among models

To compare the outbreak detection performance of the expectation-variance model with the traditional models, receiver-operator (ROC) curves were constructed for all models. ROC curves show the dependence of the mean sensitivity on the mean specificity, and the area under the ROC curve is an indicator of overall performance. The area was estimated by the trapezoidal method.

## Results

### Evaluation of specificity trends over time

**Figure 2:**
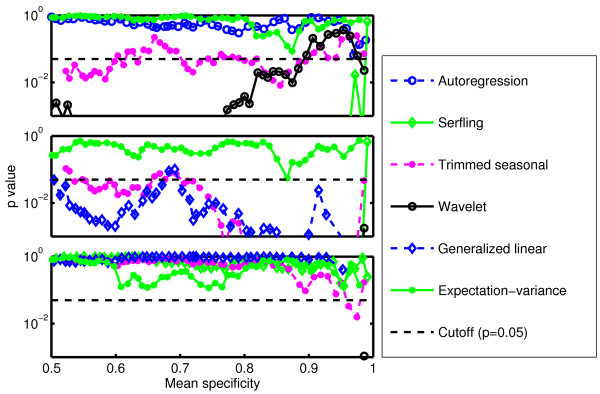
**Evaluating variability in specificity on three time scales**. Plots of *p*-values for the chi-square test over various time scales for the five comparison models over a range of mean specificity values from 0.50 to 0.99, as well as *p*-values for the expectation-variance model. Top: calendar year of study. Middle: month of year. Bottom: day of week. The expectation-variance model has a *p*-value over 0.05 for the entire range of mean specificity values for all three timescales, so the null hypothesis of constant specificity is not rejected. All plots not shown are highly significant (*p *< 0.001) for non-constancy.

**Figure 3:**
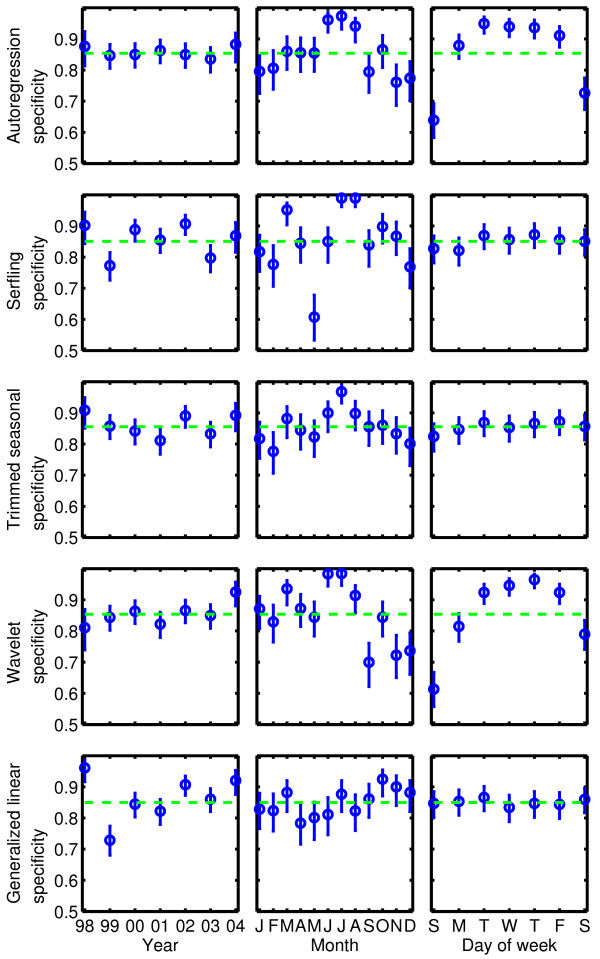
**Average specificity trends over time**. Average specificity for each calendar year, month, and day of week for the five comparison methods during the study period. Data shown were recorded for each model implemented at 85% mean specificity. Similar trends were observed for all methods at 97% mean specificity (data not shown).

As suspected, the specificity of the five standard models was not constant over time. Hypothesis testing indicated that the specificity of the Serfling, trimmed seasonal and generalized linear models varied with the study calendar year and study month (*p *< 0.05) over a range of mean specificities between 0.50 and 0.99. The autoregressive model demonstrated a variable specificity with the study month and day of the week (*p *< 0.05) for the same range of mean specificities, and the wavelet model had variable specificity (*p *< 0.05) on all three time scales (figure 2). Several trends in the specificity were apparent when the analysis was limited to particular values of mean specificity. For example, at a mean specificity of 85 percent, corresponding to approximately one false alarm each week, the autoregressive, Serfling, trimmed seasonal and wavelet models had highest specificity in June and July and low specificity during the winter months. The specificity of the autoregressive and wavelet models was highest in the middle of the week and lowest on Sunday, and the Serfling, trimmed seasonal and generalized linear models had higher specificity during certain study years (figure 3). Similar trends were observed at other mean specificity values, including 0.90, 0.95, and 0.97 (data not shown).

By contrast, the expectation-variance model specificity was constant as a function of the study year, study month, and the day of the week. Hypothesis testing resulted in a *p*-value above 0.05 for the entire range of input specificities on all three time scales, indicating that there was no evidence to suggest that the specificity was non-constant on any time scale (figure 2).

### Comparison of sensitivity and timeliness of new and traditional methods

The expectation-variance model usually outperformed traditional approaches in terms of sensitivity. The area under the expectation-variance model ROC curve was equal to or greater than that of the five comparison models for all three outbreak types (table 1).

**Table 1:** Comparative detection performance

Detection method	Flat outbreak	Linear outbreak	Spike outbreak
Autoregression	0.94	0.90	0.88
Serfling	0.93	0.88	0.89
Trimmed seasonal	0.95	0.91	0.89
Wavelet	0.93	0.87	0.86
Generalized linear	0.95	0.91	0.91
Expectation-variance	0.95	0.91	0.91

ROC curve areas for traditional and expectation-variance detection models applied to three different types of outbreaks superimposed on respiratory visits to an urban pediatric ED, August 1998 – July 2004.

The expectation-variance method also performed well in terms of earliness of detection. At a benchmark mean specificity of approximately 97 percent, it detected a seven-day outbreak consisting of five additional patients each day with a shorter lag than the autoregressive, Serfling, trimmed seasonal, and wavelet models (table 2). The expectation-variance model also had earlier detection than these models at 85 percent specificity (data not shown).

**Table 2:** Comparative detection delays

Detection method	Mean specificity	Mean sensitivity	Mean detection lag (days)
Autoregression	0.97	0.40	2.26
Serfling	0.97	0.36	2.37
Trimmed seasonal	0.97	0.42	2.26
Wavelet	0.98	0.38	2.43
Generalized linear	0.95	0.68	1.93
Expectation-variance	0.97	0.58	1.96

Mean lag in detecting outbreaks of five additional patients per day superimposed on the pediatric ED respiratory visits, August 1998 – July 2004. Detection lag calculations exclude undetected outbreaks. Hence the sensitivity of the method must be considered when interpreting the detection lag.

### Temporal sensitivity trends

**Figure 4:**
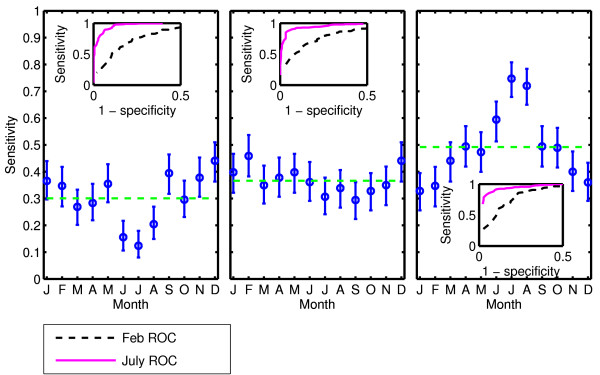
**Seasonal sensitivity trends**. Average sensitivity for each month of the study period for the autoregressive (left), trimmed seasonal (center), and expectation-variance (right) models when applied to data containing a superimposed spike outbreak of 10 additional patients during one day. Data shown were collected at a mean specificity of 97%. The sensitivity of the trimmed seasonal and autoregression models is higher during the winter than during the summer. Sensitivity is higher during the summer than during the winter for the expectation-variance model. July receiver-operator (ROC) curves lie below February ROC curves for all three models (insets). Similar trends were observed for flat and linear outbreaks.

The sensitivity of outbreak detection depends on the size and shape of an outbreak, as well as on the amount of noise in the ED utilization signal. Thus even when the specificity is held constant, it is natural for the sensitivity to vary with the season, day of the week, and trend. The ED visit signal had the least noise in the summer and the most noise in the winter (figure 4). Hence the signal-to-noise ratio was highest in the summer for any fixed type of outbreak, and the sensitivity of any reasonable detection strategy should theoretically be greater during the summer than in the winter. Summer and winter ROC curves for the expectation-variance and five comparison methods confirmed that summer sensitivity was greater than winter sensitivity when the specificity was held fixed (figure 4 insets). However, at mean specificity values of 85 and 97 percent, plots of sensitivity over time for the autoregressive, Serfling, trimmed seasonal and wavelet models showed a paradoxical increase in sensitivity to synthetic outbreaks during winter months compared to summer months (figure 4). These seemingly contradictory results occurred because the mean specificity of these four comparison models was not the actual specificity during either the summer or winter. The specificity was significantly higher during the summer, corresponding to a shift to the left along the summer ROC curve and a concomitant decline in summer sensitivity. The opposite occurred in winter. This anomaly was corrected by the expectation-variance model (figure 4), since it operated at the same specificity during all seasons. The generalized linear model exhibited variable specificity by month, but its specificity was not highest during the summer months (figure 3), and hence it also had greater summer sensitivity than winter sensitivity.

**Figure 5:**
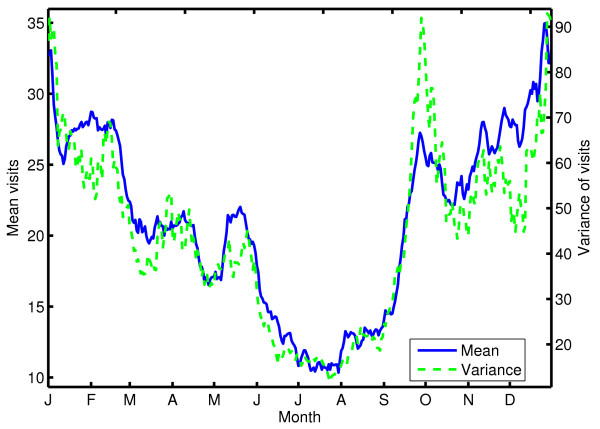
**Seasonal trends in the mean and variance of ED visits**. Mean number of ED visits (left axis, solid blue line) and mean variance in ED visits (right axis, dashed green line) as a function of the day of year. Data were smoothed using 5-day and 11-day moving averages, respectively. The ED utilization mean and variance are highest in the winter and lowest during the summer.

## Discussion

We found that the specificity of outbreak detection was not constant for five traditional algorithms. This is important because having a standardized interpretation of the statistical characteristics of an outbreak detection test, including the specificity, aids public health practitioners in making rational decisions regarding resource allocation in the event of an alarm. The positive predictive value (PPV) of an alarm, the probability that an alarm signals a real outbreak, bears directly on the priority and extent of response required. The PPV is related to the specificity by the equation

PPV=sensitivity⋅psensitivity⋅p+(1−specificity)⋅(1−p),
 MathType@MTEF@5@5@+=feaafiart1ev1aaatCvAUfKttLearuWrP9MDH5MBPbIqV92AaeXatLxBI9gBaebbnrfifHhDYfgasaacH8akY=wiFfYdH8Gipec8Eeeu0xXdbba9frFj0=OqFfea0dXdd9vqai=hGuQ8kuc9pgc9s8qqaq=dirpe0xb9q8qiLsFr0=vr0=vr0dc8meaabaqaciaacaGaaeqabaqabeGadaaakeaacqqGqbaucqqGqbaucqqGwbGvcqGH9aqpdaWcaaqaaiabbohaZjabbwgaLjabb6gaUjabbohaZjabbMgaPjabbsha0jabbMgaPjabbAha2jabbMgaPjabbsha0jabbMha5jabgwSixlabdchaWbqaaiabbohaZjabbwgaLjabb6gaUjabbohaZjabbMgaPjabbsha0jabbMgaPjabbAha2jabbMgaPjabbsha0jabbMha5jabgwSixlabdchaWjabgUcaRiabcIcaOiabigdaXiabgkHiTiabbohaZjabbchaWjabbwgaLjabbogaJjabbMgaPjabbAgaMjabbMgaPjabbogaJjabbMgaPjabbsha0jabbMha5jabcMcaPiabgwSixlabcIcaOiabigdaXiabgkHiTiabdchaWjabcMcaPaaacqGGSaalaaa@7307@

where *p *is the prior probability of an outbreak. Because the specificity of an alarm strategy affects its PPV, it is crucial to have an accurate estimate of the specificity on any particular day. Even small differences in the specificity may have a great impact on the PPV; an alarm strategy at 95 percent specificity may have a PPV nearly twice as high as the same strategy at 90 percent specificity, depending on the nature of the outbreak considered and the sensitivity of the system. A public health practitioner responding to an alarm in the first case may wish to devote twice as many resources to investigating the alarm than in the second case.

The specificity also affects the overall cost associated with a surveillance model. Let *c*_*TP*_, *c*_*FP*_, *c*_*TN *_and *c*_*FN *_denote the costs associated with true positive alarms, false positive alarms, true negatives, and false negatives, respectively. Then the expected total cost of an alarm strategy on a given day is a weighted sum of these costs:

*E*[cost] = *c*_*TP*_·sens·*p *+ *c*_*FN*_·(1 - sens)·*p *+ *c*_*FP*_·(1 - spec)·(1 - *p*) + *c*_*TN*_·spec·(1 - *p*).

Lowering the specificity contributes to the cost due to fruitlessly investigating more false positive alarms, reflected in the third summand of the equation. At a specificity of, for example, 99%, one can expect to experience a false alarm every 100 outbreak-free days. Lowering the specificity to 97% increases the false alarms to approximately once per month. The cost equation can also be used to compare two alarm methods, *A *and *B*. Strategy *A *is more cost-effective than strategy *B *if and only if the expected cost of *A *is less than that of *B*:

(sens_*A *_- sens_*B*_)(*c*_*TP*_·*p *- *c*_*FN*_·*p*) < (spec_*A *_- spec_*B*_)(*c*_*FP*_·(1 - *p*) - *c*_*TN*_·(1 - *p*)).

Thus the greater the accuracy in the estimates of the specificity and sensitivity of each method, the prior probability of an outbreak *p*, and the costs of each scenario, the more accurately a public health department can compare the cost-effectiveness of the various available surveillance methods.

It may be desirable under certain conditions to have non-constant specificity. For example, one may wish to adjust the specificity so that the PPV is constant as a function of the day of the week, season, and trend. Alternatively, a high profile event may merit special attention, requiring lower specificity surveillance to increase the sensitivity to outbreaks. The expectation-variance model is preferable to traditional models in these situations because its specificity is known more reliably than that of traditional models. Therefore the specificity can easily be adjusted with time according to public health needs. By contrast, current models operate with unknown specificity, and adjusting an unknown quantity presents a difficulty.

To understand the inability of traditional models to maintain constant specificity over time, it is useful to recast the outbreak detection problem in terms of percentiles instead of means. A perfect outbreak detection model operating at a specificity of 0.95 would output an alarm threshold equal to the 95th percentile for each day, above which an alarm would sound. More generally, a perfect model at specificity k100
 MathType@MTEF@5@5@+=feaafiart1ev1aaatCvAUfKttLearuWrP9MDH5MBPbIqV92AaeXatLxBI9gBaebbnrfifHhDYfgasaacH8akY=wiFfYdH8Gipec8Eeeu0xXdbba9frFj0=OqFfea0dXdd9vqai=hGuQ8kuc9pgc9s8qqaq=dirpe0xb9q8qiLsFr0=vr0=vr0dc8meaabaqaciaacaGaaeqabaqabeGadaaakeaadaWcaaqaaiabdUgaRbqaaiabigdaXiabicdaWiabicdaWaaaaaa@30E7@ would model the *k*th percentile. The autoregressive, Serfling, trimmed seasonal and wavelet models assume that the data have normally distributed errors with constant variance. They thus make a first approximation to this percentile by modeling the mean, to which a constant (which depends on *k*) is added. One problem with this approach is that the ED utilization signal is heteroscedastic – that is, its variance is not constant as a function of time (figure 5). In practical terms, this means that the *k*th percentile is sometimes farther from the signal mean than at other times. Hence it cannot be captured by adding a constant value to the mean. The result is that during periods of greatest ED utilization variance, such as the winter months (figure 5), the alarm thresholds of these traditional models underestimate the *k*th percentile, leading to a decreased winter specificity (figure 3). Conversely, all four models overestimate the alarm threshold during the summer months, when the ED utilization variance is lowest. In fact, neglecting the dependence of the ED visit variance on the day of week, day of year, or long-term trend when determining the alarm threshold introduces some degree of systematic error in the alarm threshold, although it may not be of sufficient magnitude to cause statistically detectable variations in the specificity.

Although the generalized linear model does not assume that the variance is constant, it does assume that the data are Poisson distributed, and consequently that the signal variance is equal to the signal mean. However, the actual signal variance is greater than the mean; the ratio ranges from approximately one to more than three during the calendar year (figure 5). The result is that during periods of high relative signal variance, the specificity of the method is also relatively high. For example, in October, both the ratio of signal variance to signal mean (figure 5) and the specificity (figure 3) are high.

Changes in specificity may also result from systematic errors in the expected number of ED visits predicted by the algorithms. For example, our implementations of the wavelet and autoregression models do not take into account day-of-week effects on the number of ED visits. Hence during high-volume days, such as Sundays, these models underestimate the expected number of visits. This in turn lowers the alarm cutoff value and the specificity compared to low-volume days such as Wednesdays. The Serfling model constrains the seasonal effects of ED utilization to a sine wave. However, the normal seasonal pattern of respiratory visits includes a spring increase that coincides with the allergy season (figure 5), which cannot be captured by a sine curve. This causes a May dip in the specificity of the Serfling model (figure 3).

In addition to the approach considered here, it may be possible to apply a generalized additive or other model to the squared residuals of a traditional algorithm. A model for the alarm threshold would then be constructed in a similar manner to the expectation-variance model. Because the specificity is affected by systematic errors in both the mean and the variance, it would be necessary to apply a statistical test to ensure that the specificity was constant.

The expectation-variance model is a general time series method which could be applied to surveillance of other syndromes and populations. Implemented here in Matlab, it could easily be imported to other platforms, and it requires minimal additional computational resources for public health departments collecting surveillance visit data. It does, however, have several limitations. While useful for modeling syndromes that are predictable functions of the trend, season, and day-of-week covariates, such as respiratory or gastrointestinal illnesses, it would have limited utility compared to simpler models for rare or sporadically occurring syndromes. The present study has evaluated the specificity, sensitivity, and timeliness of detection using a training set containing six years of data. However, this much historical data is not always available for model training. Although the algorithm is easily adapted to shorter training sets, future work is needed to assess its performance with such sets. Like other detection methods, the training data must be free of an outbreak of interest in order for the specificity estimates to be accurate. Thus the training set used in the present study would be useful for detecting anthrax, other bioterrorism events, or large influenza outbreaks due to changing viral strains, but not for reliably detecting yearly average influenza outbreaks present in the data. Like other time series methods, the model also does not take advantage of geospatial information or data streams containing different types of data.

A more subtle limitation of the expectation-variance model is that its output is a binary variable – the absence or presence of an alarm. Kleinman et al. [30] proposed an approach to temporal and spatial surveillance which instead provides the probability that an observed event would be expected in the absence of an outbreak. This approach represents a shift from statistical testing to more detailed statistical modeling techniques [31]. Although the current implementation of our method is binary, it can easily be converted to a "modeling" approach. For example, a graph of the specificity as a function of the alarm threshold corresponds to a predicted cumulative distribution function of the number of visits on any given day.

In addition to the limitations of the model, our study is limited in its analysis of sensitivity to various outbreak types. The sensitivity depends on the time series of additional outbreak patient visits, of which an infinite array of possibilities exist. In the absence of outbreak data capturing the essential features of the many diseases and syndromes that may be monitored, we have used synthetic outbreaks having simple functional forms or "canonical shapes" [32]. This makes comparisons between types of outbreaks easy to interpret. Alternatively, the response to one or more known outbreaks may be evaluated [18,33]. This approach has the advantage that the outbreaks are inherently realistic, since they are instances of true outbreaks. However, they may be highly irregular and dominated by stochastic effects. Indeed, there is no guarantee that they bear resemblance to future outbreaks of the same or other diseases. The present study offers the promising conclusion that the expectation-variance model has good comparative sensitivity for a limited number of artificial outbreaks, but more detailed study in the context of outbreaks of interest would be necessary to conclude that the model is preferable to previous models for real-world surveillance.

## Conclusion

The interpretation of alarms using current outbreak detection strategies is difficult because the specificity is extremely variable. The fluctuations in specificity are due to changes on the same time scales in the variance of the ED utilization signal. Unlike previous models, the model developed here accounts for changes with time of not only the expected number of ED visits, but also of the variance of the number of visits. It is our hope that this provides a useful method for achieving a signaling strategy with known, constant specificity, enhancing the ability of public health practitioners to interpret the meaning of an alarm.

## Authors' contributions

SW participated in the study design, carried out the study, and helped to draft the manuscript. JB, BB and KM participated in the design of the study and helped to draft the manuscript. All authors read and approved the final manuscript.

## Pre-publication history

The pre-publication history for this paper can be accessed here:


